# Editorial: Transcriptional and epigenetic control of T and innate lymphoid cell development and function

**DOI:** 10.3389/fimmu.2023.1213831

**Published:** 2023-05-11

**Authors:** Wei Xu, Mihalis Verykokakis

**Affiliations:** ^1^ Department of Immunology, School of Basic Medical Sciences, Fudan University, Shanghai, China; ^2^ Institute for Fundamental Biomedical Research, Biomedical Sciences Research Center (BSRC) Alexander Fleming, Vari, Greece

**Keywords:** transcription, epigenetics, T cells, ILC, development

The immune system is a complex network of multiple cell types and tissues whose functions should be tightly coordinated in order for the organism to mount effective responses against environmental threats and changes. All cells of the immune system derive from hematopoietic stem cells, which have numerous options to pursue different developmental pathways that lead to well-defined cell identities (1). These developmental pathways are governed by a succession of gene expression programs that promote a certain cell fate while restricting alternative lineage choices. In this Research Topic, Shin and Rothenberg provide a thorough insight into the mechanisms involved in the establishment of T cell identity as a paradigm of how gene regulatory networks may control cell fate decisions. In this model, sustained exposure of thymic progenitor cells to Notch ligands is sufficient for the initiation of the T cell program and subsequent commitment to this lineage. Installation of the T cell identity relies not only on the induction of T cell specification genes, but also on the repression of alternative non-T myeloid or innate lymphoid lineages and of the progenitor gene regulatory modules. Therefore, at least three different gene expression modules should be adjusted before precursors are able to follow the T cell pathway, which involve positive and negative feedback regulatory loops between transcription factors that help to stabilize each module.

Once T cell identity is established, these common T cell progenitors further develop into multiple T cell lineages in the thymus. The majority of cells are conventional T cells expressing TCRαβ while several unconventional T cell lineages, including γδ T cells, break off the main pathway at different stages of T cell development. Differentiation of γδ lineages begins at immature CD4^-^CD8^-^ double negative (DN) stage where the expression and signaling through the γδTCR or preTCR complex with different signal strength dictates the fate of the cell. Expression of the γδTCR depends on the activity of Eγ and Eδ enhancers, which drive germline transcription of TCR genes and subsequent V(D)J rearrangement (2, 3). Extracellular signals and transcription factors including IL-7R/STAT5 and NOTCH1/RUNX1/MYB have been shown to be important for Eγ regulation (4, 5). Rodriguez-Caparros et al. demonstrated that Eδ-dependent *Tcrd* germline transcription was regulated in the same fashion as Eγ. They showed that IL-7R signaling is essential for *Tcrd* germline transcription in DN2b and DN3a thymocytes, and optimal activation also requires Notch. STAT5 was found to be activated and recruited to Eδ in addition to Eγ. Thus, induction of Eγ and Eδ function was regulated by similar mechanisms in parallel during γδ lineage differentiation.

The activity of gene regulatory networks is strongly dependent on the availability of chromatin binding sites for the respective transcription factors. High-order chromatin architecture often poses strong barriers, which should be overcome in a spatiotemporally coordinated fashion, to enable differentiation of progenitor cells. The genome is organized in topologically associating domains (TADs), which regulate gene expression by controlling distal interactions between promoters and cis-regulatory elements. TAD boundaries are often sites of active transcription, and are characterized by the occupation of ubiquitously expressed chromatin organizers, such as CTCF and YY1 (6). However, Papadogkonas et al. indicate that tissue-specific genome organizers are also required to control TAD activity in a cell type-specific manner. They focus on the functions of SATB1, which is highly expressed in thymic CD4^+^CD8^+^ (double positive, DP) progenitors and is required for thymic T cell development and positive selection. Indeed, absence of SATB1 from DP cells disrupted the communication between promoters and distal regulatory elements, thus interrupting the proper expression of T cell master regulators and the T cell receptor locus (7).

Genomic architecture depends also on DNA methylation on cytosine, which is a dynamic process that occurs in CpG islands in promoter regions and results in transcriptional repression (8). The TET family of proteins regulates the oxidation of 5-methyl-cytosin (5mC) to 5-hydroxylmethyl-cytosin (5hmC), thus promoting gene expression. Äijö et al. showed that TET2 and TET3 regulate the thymic T cell output through binding and demethylation of the *Zbtb7b* locus (encoding for Th-POK), which is critical for the development of T and invariant Natural Killer T (iNKT) cells. The findings from Äijö et al. indicate that TET proteins regulate proper magnitude and stable expression of *Zbtb7b*, thus contributing to the establishment of the transcriptional program that maintains CD4 T cell identity.

Changes in DNA methylation and their effect on gene expression are also associated with altered T cell function in the periphery. Dysregulated CD4^+^ T helper 2 (Th2) cell function was found in both allergic rhinitis (AR) and chronic spontaneous urticaria (CSU), which often co-occur in the same patients. To understand the relationship and differences between these two types of allergic diseases, Yang et al. analyzed DNA methylation patterns and differentially expressed genes in CD4^+^ T cells between the two groups and found that the DNA methylation status was associated with activation of CD4^+^ T cells in both cases.

Eventually, cell fate determination is regulated by the function of transcription factors that integrate developmental and environmental signals to control gene expression. The aryl hydrocarbon receptor (Ahr) is a ligand-dependent transcription factor that recognizes chemicals derived from host metabolites, diet, commensal microbiota, and the environment. Ahr is broadly expressed in the cells of the immune system and contributes to a multitude of gene regulatory networks across cell types (9). Helm and Zhou summarize the functions of Ahr in T and innate lymphoid cells (ILCs), highlighting how Ahr may serve to integrate the shared pathways between adaptive and innate lymphocytes.

Taken together, the articles published in this Research Topic provide new knowledge on how gene regulatory networks contribute to the establishment of immune cell identity, which is key to the effective and timely response of the immune system against foreign insults.

## Author contributions

All authors listed have made a substantial, direct, and intellectual contribution to the work and approved it for publication. MV was supported by a grant from the Hellenic Foundation for Research and Innovation (No 486).
